# Age-dependent decrease in glutamine synthetase expression in the hippocampal astroglia of the triple transgenic Alzheimer's disease mouse model: mechanism for deficient glutamatergic transmission?

**DOI:** 10.1186/1750-1326-6-55

**Published:** 2011-07-30

**Authors:** Markel Olabarria, Harun N Noristani, Alexei Verkhratsky, José J Rodríguez

**Affiliations:** 1 Faculty of Life Sciences, The University of Manchester, Manchester, UK; 2 Institute of Experimental Medicine, ASCR, Videnska 1083, 142 20 Prague 4, Czech Republic; 3 IKERBASQUE, Basque Foundation for Science, 48011, Bilbao, Spain; 4 Department of Neurosciences, University of the Basque Country UPV/EHU, 48940, Leioa, Spain

**Keywords:** Astroglia, Alzheimer's disease, glutamine synthetase, GFAP, amyloid beta, excitotoxicity, hippocampus, plasticity

## Abstract

Astrocytes are fundamental for brain homeostasis and the progression and outcome of many neuropathologies including Alzheimer's disease (AD). In the triple transgenic mouse model of AD (3xTg-AD) generalised hippocampal astroglia atrophy precedes a restricted and specific β-amyloid (Aβ) plaque-related astrogliosis. Astrocytes are critical for CNS glutamatergic transmission being the principal elements of glutamate homeostasis through maintaining its synthesis, uptake and turnover via glutamate-glutamine shuttle. Glutamine synthetase (GS), which is specifically expressed in astrocytes, forms glutamine by an ATP-dependent amination of glutamate. Here, we report changes in GS astrocytic expression in two major cognitive areas of the hippocampus (the dentate gyrus, DG and the CA1) in 3xTg-AD animals aged between 9 and 18 months. We found a significant reduction in N_v _(number of cell/mm^3^) of GS immunoreactive (GS-IR) astrocytes starting from 12 months (28.59%) of age in the DG, and sustained at 18 months (31.65%). CA1 decrease of GS-positive astrocytes N_v _(33.26%) occurs at 18 months. This N_v _reduction of GS-IR astrocytes is paralleled by a decrease in overall GS expression (determined by its optical density) that becomes significant at 18 months (21.61% and 19.68% in DG and CA1, respectively). GS-IR N_v _changes are directly associated with the presence of Aβ deposits showing a decrease of 47.92% as opposed to 23.47% in areas free of Aβ. These changes in GS containing astrocytes and GS-immunoreactivity indicate AD-related impairments of glutamate homeostatic system, at the advanced and late stages of the disease, which may affect the efficacy of glutamatergic transmission in the diseased brain that may contribute to the cognitive deficiency.

## Introduction

The central nervous system relies on astrocytes for its correct functioning. Astroglia is critical for metabolic support to neurones by providing glucose and lactate [1,2], regulates ion environment, i.e. K^+ ^and water movements and provides reactive-oxygen-species scavengers like glutathione [3-5].

Astrocytes, as a component of the tripartite synapse, modulate neurotransmission and control the extracellular level of neurotransmitters [6-11]. Therefore, astrocytes are essential for glutamatergic transmission being key elements for "*de novo*" synthesis of glutamate and for the glutamate-glutamine cycle; which, in addition, are fundamental for the synaptic plasticity associated to cognitive processes [12,13]. The bulk of glutamate release during neurotransmission is taken up by astroglia through Na^+^-dependent glutamate transporters [9,14]. In astrocytes glutamate is converted to glutamine by glutamine synthetase (GS) [9] (which is considered astrocytic-specific enzyme although some recent studies have shown some degree of oligodendroglial and microglial GS expression under some pathological situations [15,16]). Subsequently, astrocytic glutamine is transported back to neurones for its further conversion into glutamate [9,17]. Thus, the glutamate-glutamine shuttle makes both astrocytes and GS essential for glutamatergic neurotransmission [18]. At the same time astrocytic glutamate uptake prevents glutamate excitotoxicity [9]; disturbance of astroglial-based glutamate homeostasis may lead to neurotransmitter imbalance, neuronal malfunction and death, as well as impaired cognition [18,19].

Astroglia is fundamental for the onset, progression and outcome of neuropathological processes by limiting the damage and promoting the revascularisation of the surrounding tissue through reactive astrogliosis [20-23] and by contributing to neuroinflammation by release of various pro-inflammatory factors, such as interleukins [24-26].

Alzheimer's disease (AD) is a highly malignant neurodegenerative process characterised by anomalous intraneuronal and extracellular accumulation of β-amyloid protein (Aβ) [27] and hyperphosphorilated cytoskeletal Tau protein in neurons [28]. As a consequence of this anomalous protein formation and by a yet unknown mechanism, severe loss of specific ACh neurons and synapses appear at middle and advanced stages of the disease [29]. As a result, the CNS reacts by both neuronal compensation and glial reactivity [30,31]. Recently, in a GFAP based study, we have described that the associated reactive astrogliosis observed in the triple transgenic animal model (3xTg-AD) is preceded by a generalized atrophy of astrocytes that occurs at the middle stages of the disease (9-12 months of age). Formation of the senile plaques triggers secondary astrogliosis in astrocytes associated with Aβ depositions, and the later stages of the pathology are characterized by concomitant astroglial atrophy and astrogliosis that in any case is not associated with astrocytic density alterations (12-18 months of age) [32,33]. Furthermore, and even if the two main pathological hallmarks have to be considered when studying AD, astrocytic involvement, as recently demonstrated by us [32,33], is mainly related with Aβ pathology, since astrocytes modulate extracellular volume and components and Aβ directly affects the extracellular space, while tau pathology remains intraneuronal throughout AD [28].

## Materials and methods

All animal procedures were carried out in accordance with the United Kingdom Animals (Scientific Procedures) Act of 1986 under the license from the Home Office. All efforts were made to reduce the number of animals by following the 3R's.

### Mice

Experiments were performed on male 3xTg-AD mice, which harbours the mutant genes for amyloid precursor protein (APPSwe), for presenilin 1 PS1M146 V and for tauP301 L [34,35] and their background-matching controls as described in detail previously [34-37].

### Fixation and tissue processing

Animals of different age groups (9, 12 and 18 months; n = 4-8) were anaesthetized with intraperitoneal injection of sodium pentobarbital (50 mg/kg). Mice were perfused through the aortic arch with 3.75% acrolein (25 ml, TAAB, UK) in a solution of 2% paraformaldehyde (Sigma, UK) and 0.1 M phosphate buffer (PB) pH 7.4, followed by 2% paraformaldehyde (75 ml). Brains were then removed and cut into 4 - 5 mm coronal slabs of tissue consisting of the entire rostrocaudal extent of the hippocampus, as described previously [36]. The brain sections were post-fixed in 2% paraformaldehyde for 24 hours and kept in 0.1 M PB, pH 7.4. Coronal sections of the brain were cut into 40 - 50 μm thickness using a vibrating microtome (VT1000 S, Leica, Milton Keynes, UK). Free floating brain sections in 0.1 M PB, pH 7.4 were collected and stored in cryoprotectant solution containing 25% sucrose and 3.5% glycerol in 0.05 M PB at pH 7.4. Coronal vibratome sections at levels -1.58 mm/-2.46 mm (hippocampus) posterior to Bregma, were selected for immunohistochemistry according to the mouse brain atlas of Paxinos and Franklin [38].

### Antibodies

A mouse antiserum generated against GS (anti-GS; Millipore, UK; MAB302) was used for the determination of GS positive astrocytes. A rabbit anti-GFAP IgG fraction of antiserum (Sigma-Aldrich, UK; #G9269) was used for the determination of glial cytoskeleton and comparison to GS labelling distribution. A monoclonal antibody against amyloid beta conjugated with alexa 488 (Convance, USA; SIG-39347) was employed to label neuritic plaques. The immunolabelling pattern that we obtained with these antibodies is equivalent to that obtained previously [32,39] and their specificity has also been previously demonstrated by western blot [36,40-42]. To assess for non-specific background labelling or cross reactivity between antibodies derived from different host species, a series of control experiments were performed. Omission of primary and/or secondary antibodies from the incubation solutions resulted in a total absence of target labelling (data not shown).

### Immunohistochemistry

To minimise methodological variability, sections through the dorsal hippocampus containing both hemispheres of all animals were processed at the same time using precisely the same experimental conditions. For this procedure, the vibratome sections were first incubated for 30 min in 30% methanol in 0.1 M PB and 3% hydrogenperoxide (Sigma, UK). Sections were rinsed with 0.1 M PB for 5 mins and placed in 1% sodium borohydride (Aldrich, UK) for 30 minutes. Subsequently the sections were washed with PB profusely before rinsing in 0.1 M TS for 10 minutes. Brain sections were then incubated with 0.5% albumin bovine serum (BSA, Sigma, Dorset, UK) in 0.1 M TS and 0.25% Triton X-100 (Sigma, Dorset, UK,) for 30 minutes. For the single labelling, sections were incubated for 48 hours at room temperature with primary antibody (mouse anti-GS, 1:500, cat# MAB302, Millipore, UK). The sections were rinsed in 0.1 M TS for 30 minutes and incubated in 1:200 dilution of biotinylated horse anti-mouse IgG (Vector laboratories, Peterborough, UK) for 1 hour at room temperature. Sections were rinsed in 0.1 M TS for 30 minutes, followed by incubation for 30 minutes in avidin-biotin peroxidase complex (Vetor Laboratories Ltd, Peterborough, UK). The peroxidase reaction product was visualized by incubation in a solution containing 0.022% of 3,3'diaminobenzidine (DAB, Aldrich, Gilligham, UK) and 0.003% H_2_O_2 _for 1.5 minutes as described previously [36,37]. The reaction was stopped by rinsing the tissue in 0.1 M TS for 6 minutes followed by 0.1 M PB for 15 minutes. Brain sections were permanently mounted onto gelatinized slides. Sections were then dehydrated in ascending concentration of ethanol (50, 70, 80, 90, 95 and 100%) followed by xylene; and then permanently coverslipped.

For dual immunofluorescence labelling, the sections were incubated for 48 h at room temperature in primary antibody cocktail containing: (1) mouse anti-GS (1:500) and (2) rabbit anti-GFAP (1:30,000) simultaneously. Subsequently, GS and GFAP were detected in a sequential manner on the same sections by incubation with Alexa Fluor 594-conjugated goat anti-mouse and Alexa Fluor 488-conjugated goat anti-rabbit (Invitrogen, Paisley, UK), respectively.

For triple immunofluorescence labelling, the sections were incubated for 48 h at room temperature in primary antibody cocktail containing: (1) mouse anti-GS (1:500) and (2) rabbit anti-GFAP (1:30,000) simultaneously. Subsequently, GS and GFAP were detected in a sequential manner on the same sections by incubation with Alexa Fluor 594-conjugated goat anti-mouse and Alexa Fluor 633-conjugated goat anti-rabbit (Invitrogen, Paisley, UK), respectively. Then, the sections were washed in 0.1 M TS for 30 min and incubated in 0.5% BSA in 0.1 M TS and 0.25% Triton X-100 for 30 minutes. Subsequently, sections were incubation in mouse anti-amyloid beta Alexa 488-conjugated antibody (1:2000) for 20 hours at room temperature.

Finally, in both dual and triple immunoflorescence labelling, sections were rinsed with 0.1 M TS for 30 min and permanently mounted in an aqueous medium (Vectashield; Vector laboratories, Peterborough, UK).

### GS positive cell count in hippocampus

We determined the numerical density (N_v_; #/mm^3^) of GS positive astrocytes at 9, 12 and 18 months of age in both 3xTg-AD and non-Tg mice in the DG and CA1 subfields of the hippocampus. For this, 3 - 4 representative non-consecutive coronal sections throughout the dorsal hippocampus at levels -1.70/-2.18 [38] were quantified accounting for an analyzed volume of approximately 6,000,000 μm^3 ^in the DG and 15,000,000 μm^3 ^in CA1. The specific analysed areas were the molecular layer (MoL) in the DG and all the strata of the CA1 apart from the pyramidal cell layer that is practically devoid of GS expression due to the dense packing of pyramidal somata and almost no presence of astrocytic cell bodies. GS positive astrocytes were intensely labelled against lighter background that made them easy to identify with equal chance of being counted. A single observer determined the number of GS positive astrocytes blindly; therefore, counting bias was kept to a minimum.

### Optical Density (OD) Measurement

Using computer-assisted imaging analysis (Image J 1.32j, NIH, USA), we analyzed the expression and density of GS labelling at 9, 12 and 18 months of age in both 3xTg-AD and non-Tg mice by measuring their optical density (OD) as described previously [43]. Briefly, to exclude any experimental errors and/or bias, all images were taken at constant light intensity. Optical filters were used to ensure the specificity of the signal recorded by the camera. The staining was observed throughout the thickness of the section (40 μm) using light microscopy (Nikon Eclipse 80i). No differences were observed in GS immunoreactivity throughout the thickness of the section between 3xTg-AD and non-Tg control animals; hence the changes in OD were used as measure of increased GS expression. The OD was calculated from a relative scale of intensity ranging from 0 to 255, with readout of 250 corresponding to the area with very low GS expression and 0 corresponding to the densest area of labelling. The calibration density was kept constant for measuring all section to avoid experimental variances. Sections background OD was determined from the corpus callosum (CC) that was considered as blank since GS labelling in the CC is virtually absent. GS density of the entire DG MoL and CA1 (with the exception of the pyramidal cell layer) were measured independently and a single measurement was obtained from every sub-region in each hemisphere. To analyze the change in GS density against constant control, the 255 was divided by control region (CC) and the obtained factor was multiplied by the region of interest in every given section. Inverse optical density was obtained by subtracting from the obtained background level (set at 255). Measurement of mean density were taken and averaged, after background subtraction, from each hippocampal layers in both the left and the right hemisphere of each slice. The results are shown as inverse GS density (IOD/pixel).

### GS and GFAP positive cell count in relation to Aβ plaques in CA1

Triple labelling pictures of the CA1 at 18 months of age in both 3xTg-AD and non-Tg mice were taken using confocal scanning microscopy (Leica SP2, inverted), recording layers at every 0.5 μm. Both GS and GFAP positive cells were counted separately and taking in to account their localisation regarding Aβ plaques. We considered all cells with the somata within 50 μm from the plaque border of the plaque-associated, and cell with somata positioned more distantly as cells not associated with plaques.

### Statistical analysis

Data were expressed as mean ± SEM. Unpaired t-tests were used to examine differences between 3xTg-AD and non-Tg animals at different time points and differences between away and around conditions in 3xTg-AD. Significance was accepted at p ≤ 0.05. The data were analyzed using GraphPad Prism (GraphPad Software).

## Results

**Figure 1:**
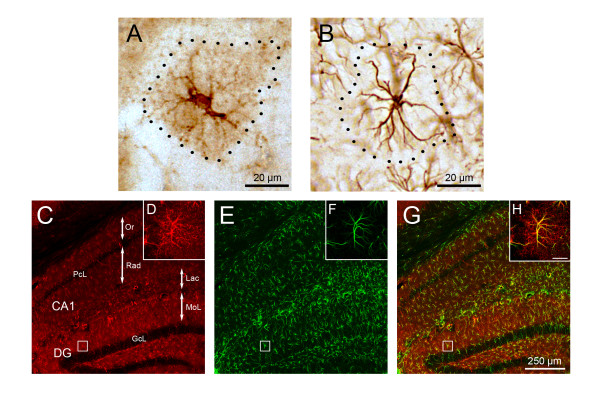
**Light and confocal micrographs of different astrocytic phenotypes according to their enzyme content and cyoskeletal component in the hippocampus of 3xTg-AD mice**. (A-B) Light microscopy images of GS (A) and GFAP (B) positive astrocytes showing their differential anatomical characteristics but similar domains. (C,E,G) Hippocampal confocal images evidencing astrocytic GS (C, red) and GFAP (E, green) expression pattern and their co-localisation (G, yellow). (D,F,H) High magnification confocal images illustrating the majoritary dual expression and co-existence of GS and GFAP (inserts D, F, H) in a representative astrocyte of the molecular layer of the DG. DG, dentate gyrus; GcL, granule cell layer; MoL, molecular layer; Lac, stratum lacunosum moleculare; Or, stratum oriens; PcL, pyramidal layer; Rad, stratum radiatum.

GS immunoreactive (GS-IR) astrocytes were widely distributed throughout the subdivisions of the hippocampus in both non-Tg and 3xTg-AD mice. This distribution was similar to the GFAP immunoreactivity in both DG and CA1 (Figure 1C, E, G, 2A). GS-IR astrocytes show typical protoplasmic morphology characterized by small round cell bodies with few primary and several secondary processes extending radially in random fashion. GFAP-IR astrocytes were characterised by primary and secondary cytoskeleton processes extending radially from the cell body and frequently co-localising with GS-IR main processes. (Figure 1A, B, D, F, H). However, and differently to GFAP-positive astrocytes, GS-IR astrocytes showed a clear and profuse labelling not only in the cell body and primary processes but also throughout the fine and thin distal processes (Figure 1A, D, 3C-F).

**Figure 2:**
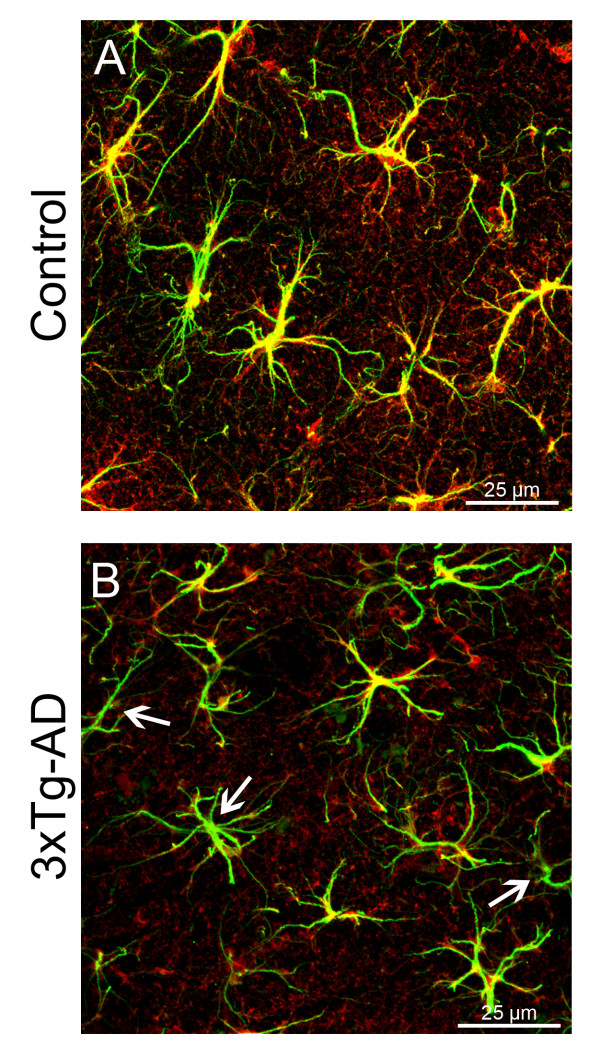
**Confocal micrographs showing GFAP (green) and GS (red) labelling in the hippocampus of either control (A) or 3xTg-AD mice (B)**. Majority of astrocytes co-express (yellow) GFAP and GS in control mice, whilst some of GFAP positive astrocytes of the 3xTg-AD mice fail to express GS (arrows).

**Figure 3:**
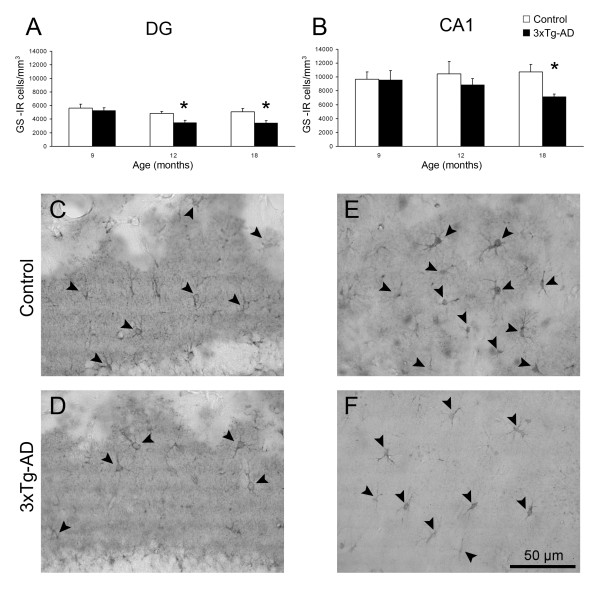
**Bar graphs illustrating GS-IR N_v _(number of cells/mm^3^) in the DG (A) and CA1 (B) of 3xTg-AD mice compared with non-Tg control animals**. Bars represent mean ± SEM. Light micrographs illustrating the difference in GS positive astrocytes (arrowheads) between non-Tg control mice and 3xTg-AD mice in either DG (C,D) or CA1 (E,F).

### GS immunoreactivity in non-Tg animals

**Figure 4:**
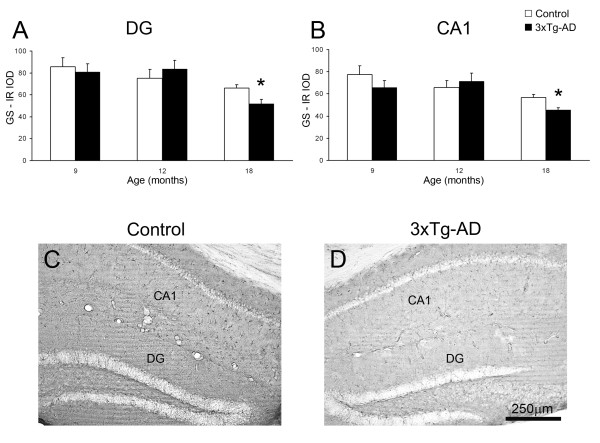
**Bar graphs showing GS content as determined by its inverted optical density (IOD) in DG (A) and CA1 (B) of 3xTg-AD mice compared with control non-Tg animals**. Bars represent mean ± SEM. Illustrative light micrographs of control mice (C) and 3xTg-AD mice (D) showing the GS expression in both the DG and CA1.

In non-Tg animals, GS-IR showed a uniform pattern in the DG and in the CA1 being constant at all age groups. The GS-IR N_v _at all ages was significantly lower in the DG compared to the CA1 (5,599 ± 603 cells/mm^3 ^vs. 9,700 ± 1,041 cells/mm^3^, 42.27%, p = 0.009; 4,852 ± 306 cells/mm^3 ^vs. 10,456 ± 1,788 cells/mm^3^, 53.59%, p = 0.021; 5,064 ± 511 cells/mm^3 ^vs. 10,727 ± 1,083 cells/mm^3^, 52.78%, p = 0.001; at 9, 12 and 18 months respectively; Figure 3A, B). The overall GS-IR, as determined by the IOD, was slightly higher in the DG at 9 and 12 months of age when compared to CA1 (10.67% and 14.38%); but just being significantly higher in the advanced age (18 months, 16.34%, p = 0.046; Figure 4A, B).

### N_v _of GS-IR astrocytes decreases in 3xTg-AD mice

From 12 months of age the 3xTg-AD mice showed a significant reduction of the N_v _of GS-IR cells in the DG (4,852 ± 306 cells/mm^3 ^vs 3,465 ± 344 cells/mm^3^; 28.59%, p = 0.016; Figure 3A) compared to the non-Tg control animals, whereas no apparent difference was found in CA1. At 18 months of age the decrease in GS-IR cell N_v _was significant in both the DG (5,064 ± 511 cells/mm^3 ^vs 3,462 ± 321 cells/mm^3^; 31.65%, p = 0.036) and in the CA1 (10,727 ± 1083 cells/mm^3 ^vs 7,159 ± 400.78 cells/mm^3^; 33.26%, p = 0.026; Figure 2, 3C-3F).

### 3xTg-AD mice exhibit a decrease in GS expression

In parallel to the decrease in the N_v _of GS-IR cells, we observed a decrease in the expression of GS in the hippocampus with no apparent regional differences, as shown by the decrease of the inverse optical density (IOD; Figure 4). The 3xTg-AD mice, when compared to non-Tg controls, showed a significantly decreased GS expression in both DG (66 ± 3 vs 52 ± 4; 21.62%; p < 0.05) and CA1 (57 ± 2 vs 46 ± 2; 19.69%; p = 0.010) at 18 months of age (Figure 4), but not at the earlier ages (Figure 4A, B).

### N_v _of GS-IR astrocytes decrease in 3xTg-AD mice is associated, although no exclusive, with Aβ plaques

**Figure 5:**
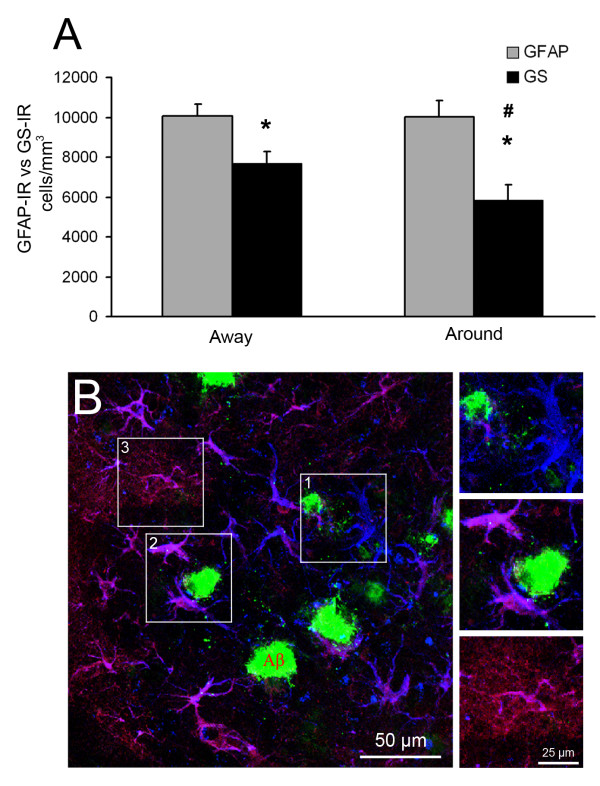
**GFAP and GS astrocytes in the hippocampus of 3xTg-AD and their relationship with Aβ plaques**. (A) Bar graph showing GFAP-IR and GS-IR astrocytes N_v _differences in the distance (away; > 50 μm) and vicinities (around; < 50 μm) of Aβ deposits. Bars represent mean ± SEM. * p ≤ 0.05 compared to correspondent GFAP-IR cell N_v_; # p ≤ 0.05 compared to GS-IR cell N_v _away. (B-E) Confocal micrographs illustrating GFAP (blue), GS (red) and Aβ (green) labelling. Several GFAP positive astrocytes in surrounding Aβ plaques (< 50 μm) lack of GS immunoreactivity (1), whilst others, co-express GFAP and GS, without the typical extended GS domain (2). (3) Astrocytes > 50 μm away from Aβ deposits co-expressing GFAP and GS in their cell bodies and main processes (pink). The distal fine processes express just GS (red).

GS-IR decrease was not homogenous throughout the CA1 parenchyma in 3xTg-AD. GS-IR N_v _was compared with GFAP-IR N_v _in either the vicinity or distant of Aβ plaques in 3xTg-AD at 18 months of age, to asses whether GS expression changes were related to Aβ plaques. No GFAP-IR N_v _related changes were observed in any of the conditions (Figure 5). However, GS-IR astrocyte N_v _was significantly diminished in CA1 areas free of Aβ (10,069 ± 572 cells/mm^3 ^vs 7,704 ± 558 cells/mm^3^; 23.49%, p = 0.010) when compared to GFAP-IR N_v _density in same areas, being this reduction more patent in the vicinities of neuritic plaques (10,314 ± 922 cells/mm^3 ^vs 5,338 ± 685cells/mm^3^; 48.24%, p = 0.002). In addition, GS-IR N_v _associated to neuritic plaques was significantly lower to GS-IR N_v _away from Aβ deposits (by 24.24%, p = 0.022).

GS-IR and GFAP-IR N_v _was almost identical in non-Tg controls, showing 99.22% of co-localisation (data not shown).

## Discussion

In the present study we analysed the functional status of astroglia in the triple transgenic AD animal model by determining the expression of the GS at different ages. Our results in this animal model demonstrate that the expression of astroglial GS in hippocampus is affected at the human equivalent advanced and late stages of AD, as indicated by a reduction in both the N_v _of GS-IR astrocytes and GS expression (as determined by IOD) in the 3xTg-AD when compared to the control animals. These changes in N_v _appear in both DG and CA1, albeit they initially occur in the DG at 12 months of age. Such reduction becomes more evident in the surroundings of Aβ plaques at 18 months of age. The GS expression is also decreased in the 3xTg-AD in both DG and CA1 but at a later age (18 months).

Astrocytes are known to be involved in most neurological diseases including AD [20,22,33]. AD is characterised by a profound cognitive impairment due to a severe loss of synapses and neurones; which is generally believed to be associated with reactive astrogliosis [28,44]. Our recent studies in the 3xTg-AD animal model, however, have found a more complex astroglial reactions in the late stages; which might show a similar pattern during human AD progression. At the early stages of the pathology astrocytes undergo generalised atrophy and down-regulation of GFAP expression, which may indicate early compromise of astrocytic homeostatic functions [23,32,33]. Several studies have previously described that GFAP abnormalities are associated with impaired glutamate homeostasis [45-48]. In a hypoxic animal model, astrocytes in the affected areas showed an abnormal GFAP cytoskeleton characterised with very short processes; which was associated with redistribution of GFAP and GLAST to the cell body, suggesting an impaired glutamate homeostasis [45]. In addition, in GFAP knock-out mice, the lack of GFAP compromised the trafficking of the GLT-1 to the cell membrane, affecting glutamate uptake [46]. Furthermore, "*in vitro*" studies have shown an inverse correlation between astroglial expression of GFAP and GS [48]. Recently has been demonstrated that, as opposed to what was thought, 3xTg-AD model shows some degree of neuronal loss as a consequence of the pathological burden [49], strengthening the relevance of this AD model and allowing the comprehension of many described synapse-related pathological events like the current one.

Studies in AD brains have shown a decrease of cortical astrocytic GS expression [50,51] that is accompanied by a "*de novo*" neuronal expression of GS as a compensatory reaction [51,52]. This direct effect on astrocytic GS expression is also present in other neurodegenerative diseases, such as Huntington's disease, which is associated with GLT-1 glutamate transporter down-regulation [53,54]. Further studies show that GS is oxidized due to Aβ in AD brains causing reduction of enzymatic activity [55] and that GS reduced expression is related to neuritic plaques in AD brain cortex [51]. On the contrary, human studies have shown increased levels of GS immunoreactivity in the prefrontal cortex in AD brains [56]. Obviously, phylogenetic proximity, tissue preservation and medical history play crucial role in the interpretation of human vs rodent data. Our current work shows that astrocytes at the late stages (plaque formation and stabilisation, 12-18 months) of AD-like pathology, in agreement with the above-mentioned studies, are functionally compromised as shown by the reduction of GS expression and therefore their capability to master the glutamate-glutamine cycle.

Thus, we could hypothesise that astrocytes fail to support neurones and control synapses from the appearance of the disease pathological burden. Furthermore, and considering astrocytic physiological function, one could consider that these changes result in an impaired glutamate homeostasis that is manifested by deficient glutamate/glutamine turnover and restricted supply of glutamine to neurones, being this failure somehow more dramatic in the proximities of Aβ plaques. Furthermore, GS deficiency could also be a consequence of impaired astrogial glutamate uptake; inducing a potential neurotoxic process by abnormal glutamate metabolism. Hence GS deficiency may reflect an altered glutamatergic neurotransmission of AD, at the advanced and late stages of the disease, which can account for a global hippocampal neurotransmitter imbalance underlaying the mnesic and cognitive impairments observed in the disease.

## Abbreviations

3xTg-AD: Triple transgenic mouse of Alzheimer's disease; ACh neurons: Acetylcholinergic neurons; AD: Alzheimer's disease; ATP: Adenosine triphosphate; CA1: Cornus ammonis 1; CC: Corpus callosum; CNS: Central nervous system; DG: Dentate gyrus; GcL Granule cell layer; GFAP: Glial fibrillary acidic protein; GLAST: Glutamate aspartate transporter; GLT-1: Glutamate transporter; GS: Glutamine synthetase; GS-IR: Glutamine synthetase immunoreactivity/-reactive; IgG: Immunoglobulin G; IOD: Inversed optical density; Lac: Stratum lacunosum moleculare; MoL: Molecular layer; N_V_: Numerical density (cell number/mm^3^); OD: Optical density; Or: Stratum oriens; PB: Phosphate buffer; PcL: Pyramidal layer; Rad: Stratum radiatum; TS: Trizma^® ^base saline.

## Competing interests

The authors declare that they have no competing interests.

## Authors' contributions

MO carried out the immunohistochemical study and contributed to the writing of the manuscript. HNN contributed to the immunhistochemical study. AV participated in the conception of the study and writing. JJR participated in the conception and design of the study and writing of the manuscript as well as coordinated the study. All authors read and approved the final manuscript.

## References

[B1] Pellerin L Bouzier-Sore AK Aubert A Serres S Merle M Costalat R Magistretti PJ Activity-dependent regulation of energy metabolism by astrocytes: an update Glia 2007 55 1251 1262 10.1002/glia.20528 17659524

[B2] Magistretti PJ Role of glutamate in neuron-glia metabolic coupling Am J Clin Nutr 2009 90 875S 880S 10.3945/ajcn.2009.27462CC 19571222

[B3] Simard M Nedergaard M The neurobiology of glia in the context of water and ion homeostasis Neuroscience 2004 129 877 896 10.1016/j.neuroscience.2004.09.053 15561405

[B4] Kofuji P Newman EA Potassium buffering in the central nervous system Neuroscience 2004 129 1045 1056 15561419 10.1016/j.neuroscience.2004.06.008 PMC2322935

[B5] Verkhratsky A Parpura V Rodriguez JJ Where the thoughts dwell: The physiology of neuronal-glial "diffuse neural net" Brain Res Rev 2011 66 133 151 10.1016/j.brainresrev.2010.05.002 20546785

[B6] Halassa MM Haydon PG Integrated brain circuits: astrocytic networks modulate neuronal activity and behavior Annu Rev Physiol 2010 72 335 355 20148679 10.1146/annurev-physiol-021909-135843 PMC3117429

[B7] Perea G Navarrete M Araque A Tripartite synapses: astrocytes process and control synaptic information Trends Neurosci 2009 32 421 431 10.1016/j.tins.2009.05.001 19615761

[B8] Wilhelmsson U Bushong EA Price DL Smarr BL Phung V Terada M Ellisman MH Pekny M Redefining the concept of reactive astrocytes as cells that remain within their unique domains upon reaction to injury Proc Natl Acad Sci USA 2006 103 17513 17518 10.1073/pnas.0602841103 17090684 PMC1859960

[B9] Danbolt NC Glutamate uptake Prog Neurobiol 2001 65 1 105 10.1016/S0301-0082(00)00067-8 11369436

[B10] Grosche J Matyash V Moller T Verkhratsky A Reichenbach A Kettenmann H Microdomains for neuron-glia interaction: parallel fiber signaling to Bergmann glial cells Nat Neurosci 1999 2 139 143 10.1038/5692 10195197

[B11] Araque A Parpura V Sanzgiri RP Haydon PG Tripartite synapses: glia, the unacknowledged partner Trends Neurosci 1999 22 208 215 10.1016/S0166-2236(98)01349-6 10322493

[B12] Kvamme E Synthesis of glutamate and its regulation Prog Brain Res 1998 116 73 85 9932371 10.1016/s0079-6123(08)60431-8

[B13] McKenna MC The glutamate-glutamine cycle is not stoichiometric: fates of glutamate in brain J Neurosci Res 2007 85 3347 3358 10.1002/jnr.21444 17847118

[B14] Kirischuk S Kettenmann H Verkhratsky A Membrane currents and cytoplasmic sodium transients generated by glutamate transport in Bergmann glial cells Pflugers Arch 2007 454 245 252 10.1007/s00424-007-0207-5 17273865

[B15] Gras G Samah B Hubert A Leone C Porcheray F Rimaniol AC EAAT expression by macrophages and microglia: still more questions than answers Amino Acids 2011 10.1007/s00726-011-0866-6 21373769

[B16] Takasaki C Yamasaki M Uchigashima M Konno K Yanagawa Y Watanabe M Cytochemical and cytological properties of perineuronal oligodendrocytes in the mouse cortex Eur J Neurosci 2010 32 1326 1336 10.1111/j.1460-9568.2010.07377.x 20846325

[B17] Deitmer JW Broer A Broer S Glutamine efflux from astrocytes is mediated by multiple pathways J Neurochem 2003 87 127 135 10.1046/j.1471-4159.2003.01981.x 12969260

[B18] Walton HS Dodd PR Glutamate-glutamine cycling in Alzheimer's disease Neurochem Int 2007 50 1052 1066 10.1016/j.neuint.2006.10.007 17141374

[B19] Choi DW Excitotoxic cell death J Neurobiol 1992 23 1261 1276 10.1002/neu.480230915 1361523

[B20] Giaume C Kirchhoff F Matute C Reichenbach A Verkhratsky A Glia: the fulcrum of brain diseases Cell Death Differ 2007 14 1324 1335 10.1038/sj.cdd.4402144 17431421

[B21] Nedergaard M Rodriguez JJ Verkhratsky A Glial calcium and diseases of the nervous system Cell Calcium 2010 47 140 149 10.1016/j.ceca.2009.11.010 20045186

[B22] Heneka MT Rodriguez JJ Verkhratsky A Neuroglia in neurodegeneration Brain Res Rev 2010 63 189 211 10.1016/j.brainresrev.2009.11.004 19944719

[B23] Verkhratsky A Olabarria M Noristani HN Yeh CY Rodriguez JJ Astrocytes in Alzheimer's disease Neurotherapeutics 2010 7 399 412 10.1016/j.nurt.2010.05.017 20880504 PMC5084302

[B24] Samland H Huitron-Resendiz S Masliah E Criado J Henriksen SJ Campbell IL Profound increase in sensitivity to glutamatergic- but not cholinergic agonist-induced seizures in transgenic mice with astrocyte production of IL-6 J Neurosci Res 2003 73 176 187 10.1002/jnr.10635 12836160

[B25] Aloisi F Care A Borsellino G Gallo P Rosa S Bassani A Cabibbo A Testa U Levi G Peschle C Production of hemolymphopoietic cytokines (IL-6, IL-8, colony-stimulating factors) by normal human astrocytes in response to IL-1 beta and tumor necrosis factor-alpha J Immunol 1992 149 2358 2366 1382099

[B26] Nedergaard M Dirnagl U Role of glial cells in cerebral ischemia Glia 2005 50 281 286 10.1002/glia.20205 15846807

[B27] Walsh DM Selkoe DJ A beta oligomers - a decade of discovery J Neurochem 2007 101 1172 1184 10.1111/j.1471-4159.2006.04426.x 17286590

[B28] Braak E Griffing K Arai K Bohl J Bratzke H Braak H Neuropathology of Alzheimer's disease: what is new since A. Alzheimer? Eur Arch Psychiatry Clin Neurosci 1999 249 Suppl 3 14 22 10654095 10.1007/pl00014168

[B29] Yankner BA Mechanisms of neuronal degeneration in Alzheimer's disease Neuron 1996 16 921 932 10.1016/S0896-6273(00)80115-4 8630250

[B30] Pekny M Nilsson M Astrocyte activation and reactive gliosis Glia 2005 50 427 434 10.1002/glia.20207 15846805

[B31] Noristani HN Olabarria M Verkhratsky A Rodriguez JJ Serotonin fibre sprouting and increase in serotonin transporter immunoreactivity in the CA1 area of hippocampus in a triple transgenic mouse model of Alzheimer's disease Eur J Neurosci 2010 32 71 79 10.1111/j.1460-9568.2010.07274.x 20576032

[B32] Olabarria M Noristani HN Verkhratsky A Rodriguez JJ Concomitant astroglial atrophy and astrogliosis in a triple transgenic animal model of Alzheimer's disease Glia 2010 58 831 838 20140958 10.1002/glia.20967

[B33] Rodriguez JJ Olabarria M Chvatal A Verkhratsky A Astroglia in dementia and Alzheimer's disease Cell Death Differ 2009 16 378 385 10.1038/cdd.2008.172 19057621

[B34] Oddo S Caccamo A Kitazawa M Tseng BP LaFerla FM Amyloid deposition precedes tangle formation in a triple transgenic model of Alzheimer's disease Neurobiol Aging 2003 24 1063 1070 10.1016/j.neurobiolaging.2003.08.012 14643377

[B35] Oddo S Caccamo A Shepherd JD Murphy MP Golde TE Kayed R Metherate R Mattson MP Akbari Y LaFerla FM Triple-transgenic model of Alzheimer's disease with plaques and tangles: intracellular Abeta and synaptic dysfunction Neuron 2003 39 409 421 10.1016/S0896-6273(03)00434-3 12895417

[B36] Rodriguez JJ Jones VC Tabuchi M Allan SM Knight EM LaFerla FM Oddo S Verkhratsky A Impaired adult neurogenesis in the dentate gyrus of a triple transgenic mouse model of Alzheimer's disease PLoS One 2008 3 e2935 10.1371/journal.pone.0002935 18698410 PMC2492828

[B37] Rodriguez JJ Jones VC Verkhratsky A Impaired cell proliferation in the subventricular zone in an Alzheimer's disease model Neuroreport 2009 20 907 912 10.1097/WNR.0b013e32832be77d 19494789

[B38] Paxinos G Franklin KBJ The mouse brain in stereotaxic coordinates 2004 Elsevier: Academic Press

[B39] Wilhelmsson U Li L Pekna M Berthold CH Blom S Eliasson C Renner O Bushong E Ellisman M Morgan TE Pekny M Absence of glial fibrillary acidic protein and vimentin prevents hypertrophy of astrocytic processes and improves post-traumatic regeneration J Neurosci 2004 24 5016 5021 10.1523/JNEUROSCI.0820-04.2004 15163694 PMC6729371

[B40] Eng LF Ghirnikar RS Lee YL Glial fibrillary acidic protein: GFAP-thirty-one years (1969-2000) Neurochem Res 2000 25 1439 1451 10.1023/A:1007677003387 11059815

[B41] Aksenov MY Aksenova MV Butterfield DA Hensley K Vigo-Pelfrey C Carney JM Glutamine synthetase-induced enhancement of beta-amyloid peptide A beta (1-40) neurotoxicity accompanied by abrogation of fibril formation and A beta fragmentation J Neurochem 1996 66 2050 2056 8780035 10.1046/j.1471-4159.1996.66052050.x

[B42] Hensley K Hall N Subramaniam R Cole P Harris M Aksenov M Aksenova M Gabbita SP Wu JF Carney JM Brain regional correspondence between Alzheimer's disease histopathology and biomarkers of protein oxidation J Neurochem 1995 65 2146 2156 7595501 10.1046/j.1471-4159.1995.65052146.x

[B43] Cordero MI Rodriguez JJ Davies HA Peddie CJ Sandi C Stewart MG Chronic restraint stress down-regulates amygdaloid expression of polysialylated neural cell adhesion molecule Neuroscience 2005 133 903 910 10.1016/j.neuroscience.2005.03.046 15927407

[B44] Alzheimer A Beiträge zur Kenntnis der pathologischen Neuroglia und ihrer Beziehungen zu den Abbauvorgängen im Nervengewebe Histologische und Histopathologische Arbeiten über die Grosshirnrinde mit besonderer Berücksichtigung der pathologischen Anatomie der Geisteskrankheiten Jena 1910 Verlag von Gustav Fischer 401 562

[B45] Sullivan SM Lee A Bjorkman ST Miller SM Sullivan RK Poronnik P Colditz PB Pow DV Cytoskeletal anchoring of GLAST determines susceptibility to brain damage: an identified role for GFAP J Biol Chem 2007 282 29414 29423 10.1074/jbc.M704152200 17684014

[B46] Hughes EG Maguire JL McMinn MT Scholz RE Sutherland ML Loss of glial fibrillary acidic protein results in decreased glutamate transport and inhibition of PKA-induced EAAT2 cell surface trafficking Brain Res Mol Brain Res 2004 124 114 123 15135219 10.1016/j.molbrainres.2004.02.021

[B47] Pekny M Eliasson C Siushansian R Ding M Dixon SJ Pekna M Wilson JX Hamberger A The impact of genetic removal of GFAP and/or vimentin on glutamine levels and transport of glucose and ascorbate in astrocytes Neurochem Res 1999 24 1357 1362 10.1023/A:1022572304626 10555775

[B48] Weir MD Thomas DG Effect of dexamethasone on glutamine synthetase and glial fibrillary acidic protein in normal and transformed astrocytes Clin Neuropharmacol 1984 7 303 306 10.1097/00002826-198412000-00005 6150761

[B49] Fuhrmann M Bittner T Jung CK Burgold S Page RM Mitteregger G Haass C LaFerla FM Kretzschmar H Herms J Microglial Cx3cr1 knockout prevents neuron loss in a mouse model of Alzheimer's disease Nat Neurosci 2010 13 411 413 10.1038/nn.2511 20305648 PMC4072212

[B50] Le Prince G Delaere P Fages C Lefrancois T Touret M Salanon M Tardy M Glutamine synthetase (GS) expression is reduced in senile dementia of the Alzheimer type Neurochem Res 1995 20 859 862 10.1007/BF00969698 7477679

[B51] Robinson SR Neuronal expression of glutamine synthetase in Alzheimer's disease indicates a profound impairment of metabolic interactions with astrocytes Neurochem Int 2000 36 471 482 10.1016/S0197-0186(99)00150-3 10733015

[B52] Robinson SR Changes in the cellular distribution of glutamine synthetase in Alzheimer's disease J Neurosci Res 2001 66 972 980 10.1002/jnr.10057 11746426

[B53] Lievens JC Woodman B Mahal A Spasic-Boscovic O Samuel D Kerkerian-Le Goff L Bates GP Impaired glutamate uptake in the R6 Huntington's disease transgenic mice Neurobiol Dis 2001 8 807 821 10.1006/nbdi.2001.0430 11592850

[B54] Jacob CP Koutsilieri E Bartl J Neuen-Jacob E Arzberger T Zander N Ravid R Roggendorf W Riederer P Grunblatt E Alterations in expression of glutamatergic transporters and receptors in sporadic Alzheimer's disease J Alzheimers Dis 2007 11 97 116 17361039 10.3233/jad-2007-11113

[B55] Castegna A Aksenov M Aksenova M Thongboonkerd V Klein JB Pierce WM Booze R Markesbery WR Butterfield DA Proteomic identification of oxidatively modified proteins in Alzheimer's disease brain. Part I: creatine kinase BB, glutamine synthase, and ubiquitin carboxy-terminal hydrolase L-1 Free Radic Biol Med 2002 33 562 571 10.1016/S0891-5849(02)00914-0 12160938

[B56] Burbaeva G Boksha IS Tereshkina EB Savushkina OK Starodubtseva LI Turishcheva MS Glutamate metabolizing enzymes in prefrontal cortex of Alzheimer's disease patients Neurochem Res 2005 30 1443 1451 10.1007/s11064-005-8654-x 16341942

